# Astragaloside IV Improves Cognitive Impairment in Alzheimer’s Mice by Alleviating Neuron PANoptosis

**DOI:** 10.3390/ijms27083508

**Published:** 2026-04-14

**Authors:** Weihai Wang, Yidong Zhao, Zheyuan Li, Yiting Lv, Zhikang Xu, Baojie Qi, Jing Yin, Chunsheng Wang

**Affiliations:** College of Life Science, Northeast Forestry University, Harbin 150040, China

**Keywords:** Alzheimer’s disease, astragaloside IV, PANoptosis, neurons, 3×Tg-AD mice

## Abstract

Alzheimer’s disease (AD) is a neurodegenerative disorder for which no effective treatments are currently available. PANoptosis is a coordinated cell death pathway involving pyroptosis, apoptosis, and necroptosis. Astragaloside IV (AS-IV) is a bioactive saponin derived from Astragalus membranaceus. Behavioral performance was evaluated using the Morris water maze and open field tests, while neuronal damage was assessed by Nissl staining. The expression levels of Aβ, IL-18, and PANoptosis-related proteins were analyzed by Western blot. Immunofluorescence was performed to assess the co-localization of PANoptosis-associated proteins with neurons in the hippocampal region. In addition, the effects of AS-IV on the expression of PANoptosis-related proteins were examined in Aβ-induced HT22 cells. AS-IV improved spatial memory performance and alleviated anxiety-like behaviors in AD mice. Furthermore, AS-IV treatment significantly reduced Aβ protein levels and attenuated neuronal loss in the hippocampus. Key markers of PANoptosis were downregulated following AS-IV treatment. Immunofluorescence revealed strong co-localization between PANoptosis-associated proteins and neurons. In vitro, AS-IV also inhibited the Aβ-induced upregulation of PANoptosis-related proteins in HT22 cells. Collectively, these results indicate that AS-IV exerts neuroprotective effects in AD models, which may be associated with reduced Aβ protein deposition, attenuated neuronal loss, and the regulation of PANoptosis-related proteins in the hippocampus.

## 1. Introduction

Alzheimer’s disease (AD) is a neurodegenerative disorder and the primary cause of dementia, representing a major source of disability in the elderly. Clinically, AD is characterized by memory decline, speech impairment, altered consciousness, impaired judgment, and other cognitive deficits, often accompanied by behavioral and psychological symptoms. These manifestations collectively impose a significant burden on both individuals and society [1].

Macroscopically, AD brains exhibit moderate cortical atrophy, predominantly within the frontotemporal association cortex [2,3]. This is evidenced by widened sulci and shrunken gyri in the frontal and temporal lobes, alongside notable hippocampal volume reduction [4,5]. Another common macroscopic feature is the loss of neuromelanin pigment in the locus coeruleus [6]. At the molecular level, AD is defined by two hallmark pathologies: neurofibrillary tangles (NFTs) and amyloid plaques [7,8]. NFTs arise from the hyperphosphorylation of tau protein, leading to microtubule aggregation. Amyloid plaque formation results primarily from the aberrant enzymatic cleavage of the amyloid precursor protein (APP), generating toxic oligomers that aggregate into insoluble plaques [9].

Activated microglia colocalize with these amyloid plaques and internalize Aβ [10]. This process activates the NLRP3 inflammasome, promoting the maturation and secretion of pro-inflammatory cytokines IL-1β and IL-18. This inflammatory cascade exacerbates neuroinflammation [11,12,13], drives irreversible neuronal death in proximate regions, and ultimately amplifies AD pathology [14,15].

Following exposure to glial-mediated inflammatory responses, neurons can undergo apoptosis [16], pyroptosis, and necroptosis [17]. However, growing evidence indicates significant crosstalk and coordination among these three cell death pathways. The concurrent activation of these programmed cell death modalities within a single cell is termed PANoptosis [18,19,20]. Substantial evidence supports a causal relationship between PANoptosis and AD onset, with differential expression profiles of key PANoptosis genes observed in AD brain tissue [21]. The expression level of ZBP1, a core sensor in PANoptosis, is positively correlated with AD symptom severity. Consequently, targeting PANoptosis may present a viable therapeutic strategy for AD [22,23].

AS-IV exhibits diverse pharmacological properties, including anti-inflammatory, antioxidant, anti-apoptotic, hypoglycemic, and organ-protective effects [24,25,26,27]. Notably, AS-IV has been shown to alleviate AD symptoms. Its mechanisms involve targeting EGFR and IL-1β to modulate microglial activity [28]. In LPS-induced BV2 microglia, AS-IV significantly reduces mRNA levels of pro-inflammatory factors (IL-1β, TNF-α) and inflammation-related enzymes (iNOS, COX-2) by inhibiting IκB and p65 phosphorylation, thereby suppressing NF-κB pathway activation [29]. Furthermore, AS-IV counteracts β-amyloid oligomer-induced memory impairment and hippocampal neuron apoptosis by activating PPARγ, which enhances the expression of brain-derived neurotrophic factor (BDNF) and the phosphorylation of its receptor, TrkB [30]. Collectively, these studies demonstrate that AS-IV mitigates AD pathology through multi-target mechanisms.

Based on the established role of AS-IV in ameliorating AD and the unresolved question of its effect on PANoptosis, this study hypothesizes that AS-IV alleviates cognitive impairment by inhibiting neuronal PANoptosis in AD model mice. To test this hypothesis, we treated 3×Tg-AD mice with AS-IV and observed improved learning and memory function. At the cellular and molecular levels, we detected reduced expression of neuronal PANoptosis markers (CASP3, MLKL, GSDMD) in the hippocampus. Integrated with analysis of AD pathological markers, these findings clarify that AS-IV confers neuroprotection and exerts therapeutic effects in AD by inhibiting the neuronal PANoptosis pathway, providing experimental evidence to support its clinical application.

## 2. Results

### 2.1. AS-IV Improves Learning, Memory, and Spatial Exploration in 3×Tg-AD Mice

**Figure 1:**
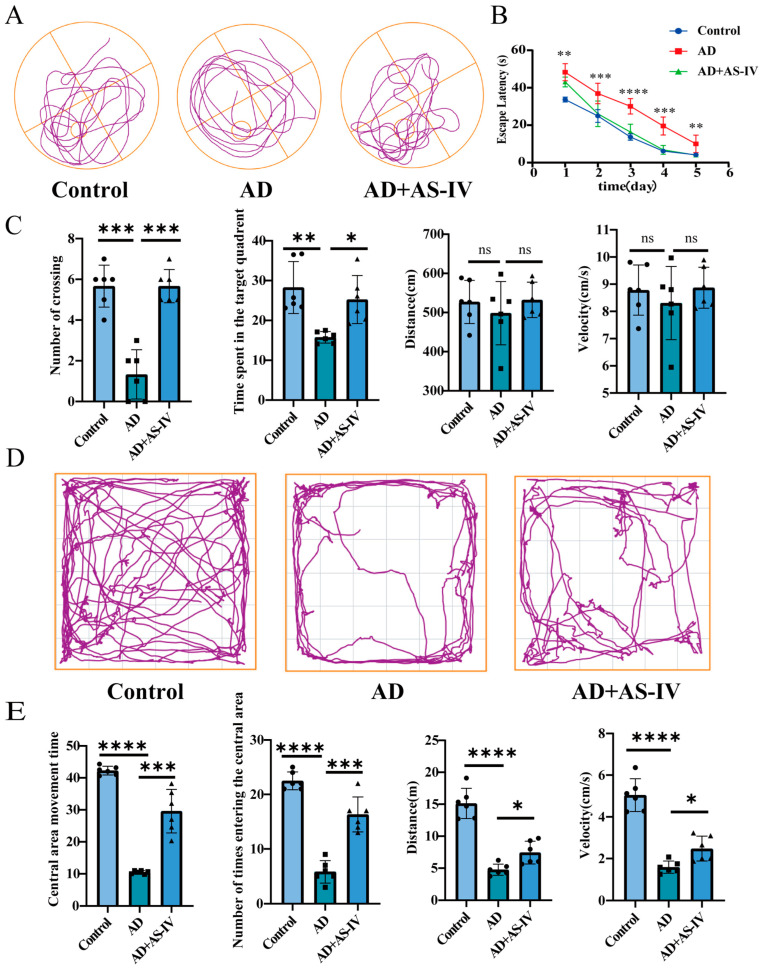
The effects of AS-IV on cognitive function and anxiety-like behaviors in AD mice. (**A**) Representative movement trajectories of mice in the Morris water maze during the probe trial on the final test day, with the platform removed. (**B**) Escape latency across training days. (**C**) Quantitative analysis of the number of platform crossings, time spent in the target quadrant, total distance traveled, and average swimming speed during the probe trial. (**D**) Representative movement trajectories in the open field test, with the center zone defined as the central 2 × 2 area of the arena. (**E**) Quantitative analysis of the number of center zone entries, time spent in the center zone, total distance traveled, and average locomotor speed. For all experiments, *n* = 6. Data are presented as the mean ± SEM; * *p* < 0.05, ** *p* < 0.01, *** *p* < 0.001, and **** *p* < 0.0001.

To determine the optimal concentration of AS-IV for alleviating AD in mice, we administered AS-IV at different concentrations (12.5, 25, 50 mg/kg) and conducted Morris water maze and open field tests. Behavioral assessments indicated that only treatment with 50 mg/kg AS-IV significantly alleviated cognitive dysfunction in both tests (Figures S1 and S2). We therefore selected this dose for subsequent experiments. Trajectory maps from the water maze test indicated that mice in the AS-IV group moved more frequently towards the target quadrant compared to those in the AD group (Figure 1A). The escape latency (i.e., the time required to locate the submerged platform) of the AS-IV group decreased significantly over training days. Notably, the reduction in escape latency was significantly greater in the AS-IV group than in the AD group (Figure 1B). Additionally, the AS-IV group exhibited more platform crossings and spent more time in the target quadrant than the AD group, with no significant differences in total movement distance or speed between groups (Figure 1C). This suggests that the observed group differences are not due to differences in movement distance. These results suggest that AS-IV may enhance memory consolidation to improve cognitive dysfunction.

In the open field test, AD mice exhibited significantly decreased total distance traveled and average locomotor speed compared to the control group, deficits that were effectively reversed by AS-IV treatment (Figure 1E). Furthermore, the AS-IV group showed significantly more entries into the central area and spent more time there than AD mice (Figure 1D,E). Together, these data indicate that AS-IV treatment successfully rescues general locomotor activity, reduces anxiety-like behavior, and promotes exploratory desire. These findings demonstrate that continuous administration of 50 mg/kg AS-IV not only enhances spatial learning and memory in AD mice but also alleviates anxiety-like behaviors and motor-exploratory deficits, providing key behavioral evidence for the efficacy of AS-IV in treating cognitive and behavioral dysfunction.

### 2.2. AS-IV Reduces Neuronal Damage and Aβ Protein Expression in the Hippocampus of 3×Tg-AD Mice

**Figure 2:**
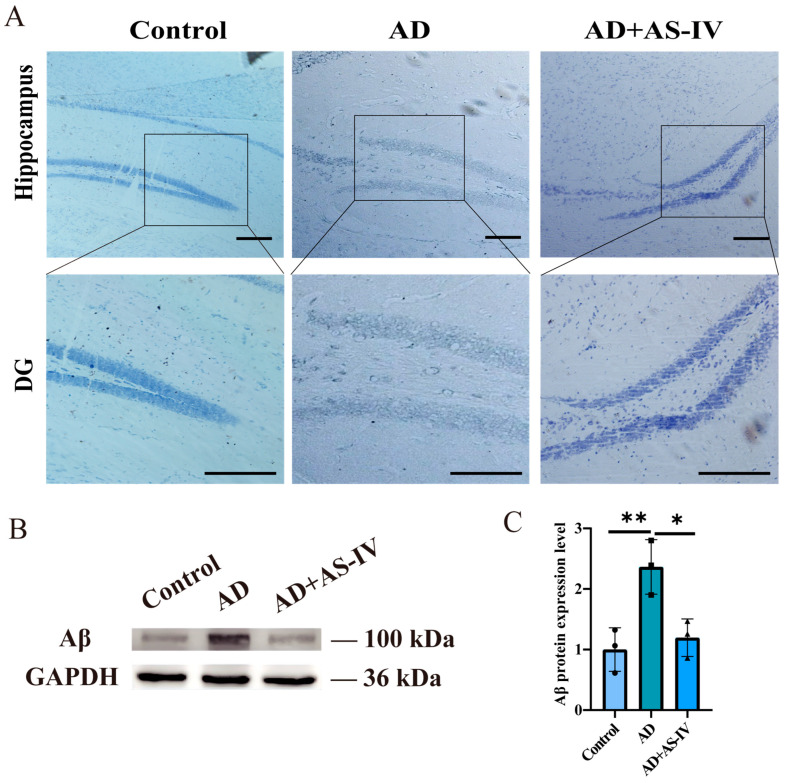
The effects of AS-IV on neuronal cells in the hippocampus of AD mice. (**A**) Nissl staining of neurons in the dentate gyrus (DG) of the hippocampus from control, AD, and AD + AS-IV groups (paraffin sections, 7 μm thickness). Scale bar = 100 μm. (**B**) Representative Western blot images of Aβ protein expression levels, with data normalized to GAPDH. (**C**) Quantitative analysis of Aβ expression levels. For all experiments, *n* = 3 (biologically independent animals per group). Quantitative data are presented as the mean ± SEM. * *p* < 0.05, ** *p* < 0.01.

In AD, neuronal damage is characterized by reduced neuronal density, disorganized cellular arrangement, and a loss of Nissl bodies. As shown in Figure 2A, neurons in the control group were closely arranged and exhibited abundant, deeply stained Nissl bodies within the cytoplasm. In contrast, the AD group displayed prominent pathological alterations, including neuronal atrophy, nuclear pyknosis, marked reduction or loss of Nissl bodies, and vacuolar degeneration of the tissue. Treatment with AS-IV markedly attenuated these pathological changes. Neurons in the AS-IV group showed a more organized arrangement, reduced vacuolation, and preserved cellular morphology, resembling that observed in the control group, indicating that AS-IV alleviates neuronal damage in the hippocampus of AD mice. At the molecular level, Western blot analysis revealed a significant increase in Aβ protein expression in the hippocampus of AD mice compared with controls, which was significantly reduced following AS-IV treatment (Figure 2B,C).

Collectively, these results demonstrate that AS-IV ameliorates neuronal pathological damage in AD mice, accompanied by a reduction in hippocampal Aβ protein levels.

### 2.3. AS-IV Inhibits the Expression of PANoptosis-Related Proteins in the Hippocampus of 3×Tg-AD Mice

**Figure 3:**
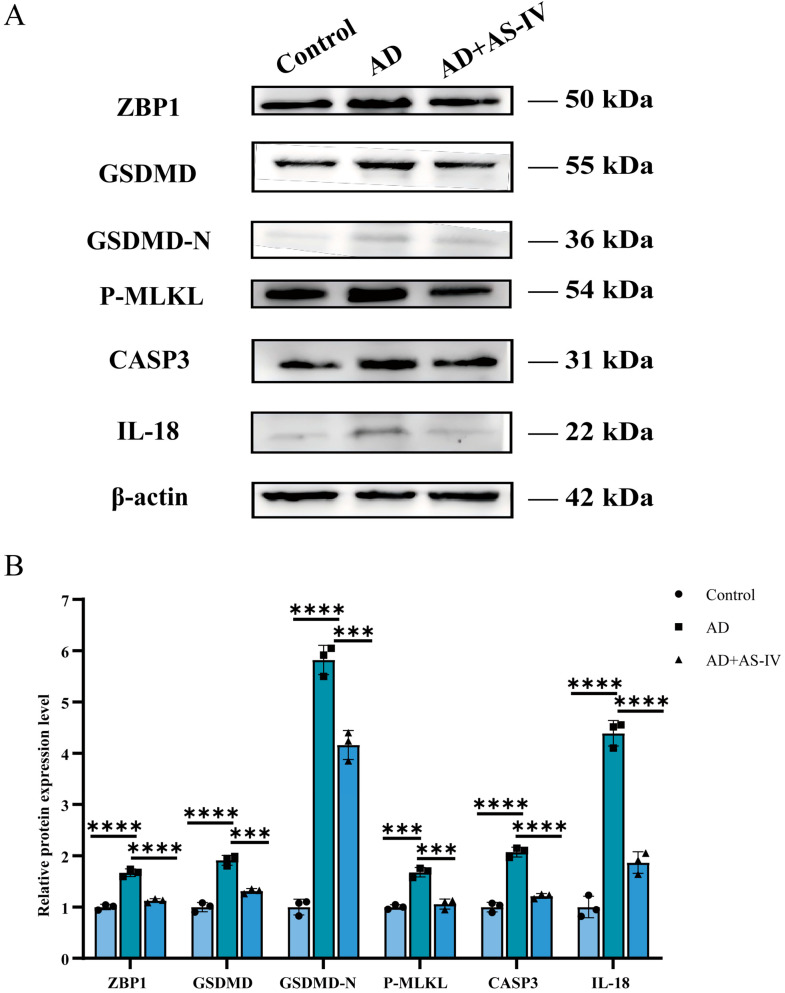
Expression levels of PANoptosis-related proteins. (**A**) Quantitative analysis of protein expression levels (ZBP1, GSDMD, GSDMD-N, p-MLKL, CASP3, IL-18), normalized to β-actin. The displayed bands are representative and may derive from different blots or experimental repeats, each target protein is explicitly matched with its corresponding loading control from the same gel. (**B**) Statistical analyses for each protein. For all experiments, *n* = 3 (biologically independent animals per group). Data are presented as the mean ± SEM; *** *p* < 0.001, and **** *p* < 0.0001.

To determine whether AS-IV modulates pathological features associated with programmed cell death in the hippocampus of AD mice, we examined the expression of key proteins involved in multiple cell death pathways that together constitute PANoptosis, including the regulatory and execution proteins ZBP1, CASP3, GSDMD, GSDMD-N, and phosphorylated MLKL (p-MLKL), as well as the inflammation-related cytokine IL-18. Western blot analysis revealed that, compared with the control group, the expression levels of PANoptosis core proteins were significantly upregulated in the hippocampus of AD mice, accompanied by a marked increase in IL-18 expression. Treatment with AS-IV markedly reduced the expression levels of all examined proteins (Figure 3A,B).

Collectively, these results demonstrate that AS-IV treatment significantly reduces the expression of core PANoptosis proteins and alleviates the associated inflammatory response in the hippocampus of AD mice.

### 2.4. AS-IV Alleviates PANoptosis of Neurons in the Hippocampal Region

**Figure 4:**
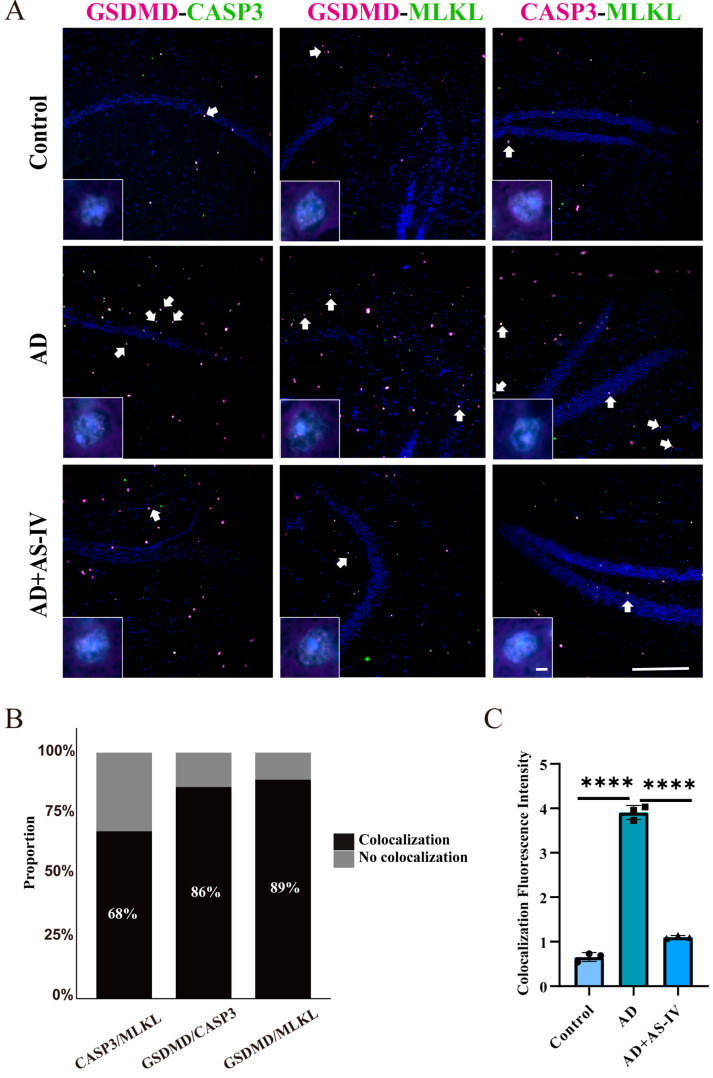
Immunofluorescence analysis of PANoptotic protein co-localization. (**A**) Representative immunofluorescence images showing colocalization of CASP3 (green) with GSDMD (purple), GSDMD (purple) with MLKL (green), and CASP3 (purple) with MLKL (green), from left to right. Notably, to illustrate the widespread and global nature of PANoptosis pathology across the AD brain, the representative high-magnification images for different protein pairs were intentionally sampled from various vulnerable subfields within the hippocampal formation (e.g., CA1, CA3, and DG), rather than being restricted to a single micro-region. Arrows indicate representative signals of fluorescence colocalization. (**B**) The proportion of cells exhibiting PANoptotic protein co-localization in the AD group. (**C**) Signal intensity of co-localized proteins in the AD group (paraffin sections, 5 μm thickness). Scale bar = 100 μm. Data are presented as the mean ± SEM; **** *p* < 0.0001.

To further confirm the activation of PANoptosis signaling in the hippocampus of AD mice, we performed dual immunofluorescence colocalization analyses targeting three core execution proteins of PANoptosis—CASP3, MLKL, and GSDMD (Figure 4A and Figures S3–S5). Strong colocalization signals for CASP3/GSDMD, GSDMD/MLKL, and CASP3/MLKL were observed in the hippocampus of AD mice, indicating the simultaneous activation of multiple cell death execution pathways, which represents a characteristic feature of PANoptosis (Figure 4B). Notably, AS-IV treatment significantly reduced both the immunofluorescence intensity and the extent of colocalization of these PANoptotic proteins (Figure 4B,C), consistent with the decreased expression of PANoptosis-related proteins detected by Western blot analysis.

**Figure 5:**
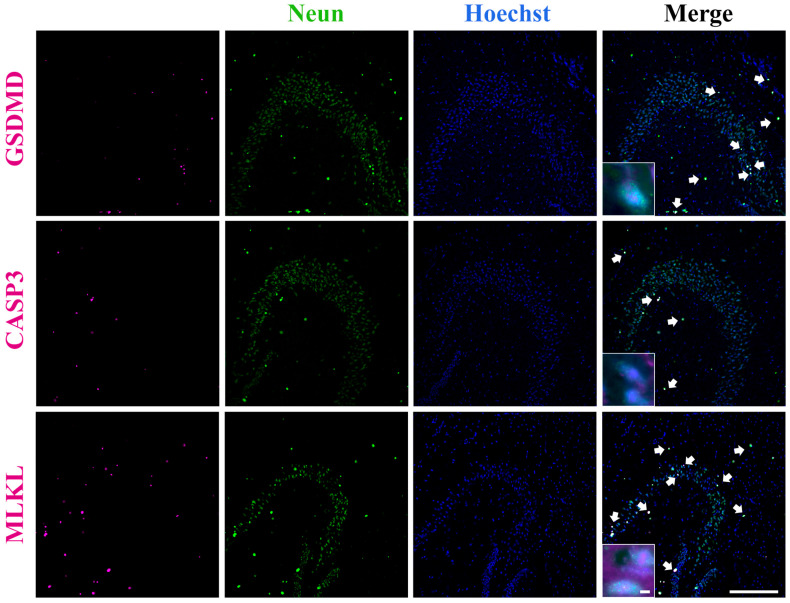
Neuronal localization of PANoptotic proteins in the hippocampus of AD mice. Representative immunofluorescence images showing colocalization of the PANoptosis-related proteins CASP3, MLKL, and GSDMD (purple) with the neuronal marker NeuN (green) (paraffin sections, 5 μm thickness). Arrows indicate representative signals of fluorescence colocalization. Scale bar = 100 μm.

Given that neuronal loss is a core pathological hallmark of AD, we next sought to determine whether PANoptosis-associated signaling occurs within neurons. To address this possibility, we examined the colocalization of PANoptosis-related proteins with the neuronal marker NeuN. Immunofluorescence analysis revealed widespread colocalization of CASP3, MLKL, and GSDMD with NeuN-positive cells in the hippocampus of AD mice (Figure 5), indicating that PANoptosis-associated signals are broadly present in neurons in the AD group.

Collectively, these findings demonstrate that PANoptosis-related signaling pathways are activated in hippocampal neurons under AD pathological conditions, and that AS-IV treatment effectively attenuates this neuron-associated PANoptotic signature.

### 2.5. AS-IV Inhibits the Expression of PANoptosis-Related Proteins in HT22

To investigate whether AS-IV modulates Aβ-induced pathological responses at the cellular level, BV2 microglial cells and HT22 neuronal cells were exposed to Aβ oligomers in the presence or absence of AS-IV. CCK-8 assays identified 1 μM and 2 μM AS-IV as the optimal treatment concentrations for BV2 and HT22 cells, respectively (Figure S6).

**Figure 6:**
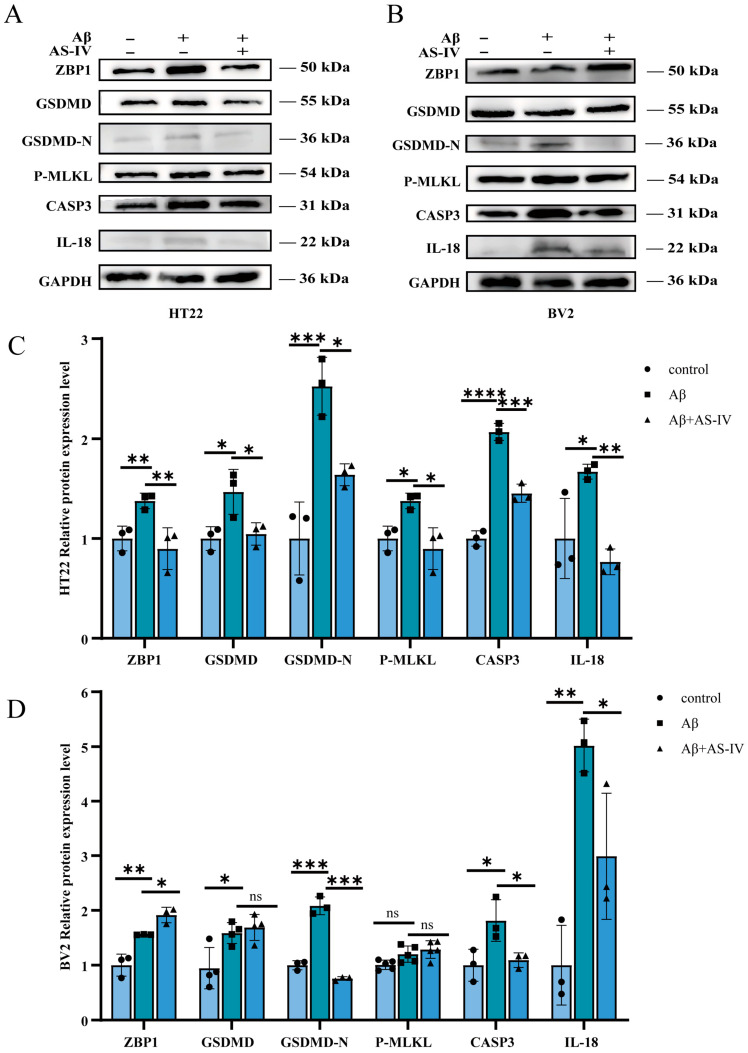
Expression of PANoptosis-related proteins in HT22 and BV2 cells. (**A**) Representative Western blot images of PANoptosis-related proteins (ZBP1, GSDMD, GSDMD-N, p-MLKL, CASP3, and IL-18) in HT22 cells. The displayed bands are representative and may derive from different blots or experimental repeats; each target protein is explicitly matched with its corresponding loading control from the same gel. (**B**) Representative Western blot images of these proteins in BV2 cells. (GAPDH is shown as the representative internal control for visual clarity.) (**C**) Quantitative analysis of protein expression levels normalized to the corresponding internal control (β-actin or GAPDH) in HT22 cells. (**D**) Quantitative analysis of protein expression levels normalized to the corresponding internal control (β-actin or GAPDH) in BV2 cells. For all experiments, *n* = 3 biologically independent samples per group. Quantitative data are presented as mean ± SEM. * *p* < 0.05, ** *p* < 0.01, *** *p* < 0.001, **** *p* < 0.0001.

Western blot analysis showed that Aβ oligomer stimulation markedly upregulated the expression of multiple core execution proteins associated with PANoptosis in HT22 neuronal cells, including ZBP1, GSDMD, GSDMD-N, CASP3, and phosphorylated MLKL (p-MLKL), accompanied by a significant increase in the inflammation-related cytokine IL-18 (Figure 6A). AS-IV treatment significantly reduced the expression levels of all these proteins (Figure 6C), indicating that AS-IV effectively suppresses Aβ-induced activation of PANoptosis-related signaling in neurons, along with concomitant attenuation of IL-18–associated inflammatory responses.

In contrast, Aβ stimulation also resulted in a significant increase in IL-18 expression in BV2 microglial cells; however, PANoptosis core execution proteins did not exhibit a broad or coordinated pattern of expression changes (Figure 6B). AS-IV treatment markedly reduced IL-18 expression in BV2 cells, while its effects on PANoptosis-related execution proteins were relatively limited (Figure 6D).

Collectively, these findings indicate that under Aβ-stimulated conditions, PANoptosis-associated molecular alterations are more prominent in neurons, whereas microglial responses are primarily characterized by regulation of the inflammatory cytokine IL-18, with insufficient evidence to support the occurrence of a canonical PANoptosis process in microglial cells.

## 3. Discussion

AD is a progressive illness characterized by gradual neuronal loss, which is the main cause of worsening cognitive and behavioral impairments [31]. Despite extensive research, effective treatments remain elusive due to the complexity of AD pathogenesis. Therefore, exploring its pathogenic mechanisms and developing effective therapeutics is urgent.

In the present study, AS-IV significantly improved cognitive and memory performance in AD mice and effectively alleviated structural brain damage (Figure 1 and Figure 2). Given the well-documented anti-inflammatory properties of AS-IV, we initially sought to explore whether this bioactivity persists within our specific AD model [28,29,30,32]. Consistent with previous reports, we observed a marked reduction in the expression of the pro-inflammatory cytokine IL-18 (Figure 3A,B). While a single cytokine measurement is insufficient to draw definitive conclusions regarding broad neuroinflammatory networks, this targeted reduction indicates that AS-IV treatment may possess potential anti-inflammatory capacity in the context of AD.

Beyond these foundational behavioral and anti-inflammatory improvements, our study provides new insights into a broader spectrum of molecular events modulated by AS-IV. While previous studies have largely attributed its neuroprotective benefits to isolated anti-inflammatory or anti-apoptotic pathways, our research introduces the concept of PANoptosis to offer a more holistic perspective on neuronal survival [18,28,29,30]. Unlike prior research that focused on singular signaling cascades, our findings highlight that AS-IV can concurrently suppress multiple PANoptosis-associated molecular markers. This shifts the focus from isolated pathways to a broader multi-pathway framework, suggesting that AS-IV exerts its robust efficacy by simultaneously attenuating concurrent cell death-related processes. This perspective offers a novel lens for understanding the complex neuronal loss observed in AD and provides a more comprehensive framework for exploring the pharmacological potential of AS-IV.

Recent studies suggest that PANoptosis may occur in AD, but conclusive evidence has been lacking [21,33]. In our study, immunofluorescence colocalization of core PANoptosis proteins revealed a high degree of spatial overlap among three key executioners in the hippocampus of AD mice (Figure 4). While these observations suggest a potential link between PANoptosis and AD pathology, they primarily reflect correlative changes rather than direct mechanistic interactions. Given the significant neuronal loss during the progression of AD, we further performed colocalization analysis of PANoptosis proteins with neuronal markers [17]. The results showed extensive colocalization of all three PANoptosis core proteins with neuronal markers in AD mice (Figure 5), hinting at the possibility that PANoptosis-related molecular events may occur in neurons following the onset of AD. Concurrently, our in vitro data obtained from HT22 neuronal cells demonstrate that AS-IV attenuates the Aβ-induced upregulation of PANoptosis-associated proteins. These findings support the hypothesis that AS-IV might ameliorate AD-related pathological changes by modulating these molecular alterations, thereby potentially reducing neuronal damage.

PANoptosis can be initiated by various sensors, such as ZBP1, AIM2, and RIPK1 [34,35]. Upon activation, these sensors promote the formation of a PANoptosome complex, ultimately executing PANoptosis [23]. Among these, ZBP1 has been implicated in AD neuroinflammation, where its activation is thought to exacerbate inflammatory damage in the AD brain. Studies show that silencing the ZBP1 gene reduces cellular damage, oxidative stress, and inflammation in AD neurons [22]. In the present study, the concurrent suppression of multiple cell death-related markers observed in AS-IV–treated AD mice exhibits a pattern similar to the characteristic features of ZBP1-mediated PANoptosis [36]. Accordingly, we speculate that the neuroprotective effects of AS-IV in AD could potentially be linked to the downregulation of ZBP1 expression and the subsequent moderation of the ZBP1/PANoptosis signaling pathway. These preliminary findings raise the possibility that ZBP1 could serve as a potential biomarker for assessing AD severity or therapeutic responsiveness in the future.

Sex differences have increasingly been recognized as important factors influencing the incidence, progression, and pathological characteristics of AD [37,38]. Epidemiological and clinical studies indicate that women have a higher risk of developing AD, which is partially attributed to menopause-associated hormonal changes and the neuroprotective effects of estrogen. In addition, female patients often exhibit more pronounced hippocampal atrophy and a greater neuropathological burden compared with male patients [39]. In the present study, experimental animals were randomly assigned to groups with comparable sex distributions. Preliminary behavioral assessments did not reveal significant sex-related differences, and therefore subsequent analyses were not stratified by sex. However, the absence of behavioral differences does not preclude the possibility of sex-specific pathological alterations. Future studies incorporating sex-stratified analyses and targeted experimental designs will be necessary to further elucidate the role of sex differences in AD pathogenesis and therapeutic responses.

While this integrated perspective offers novel insights, our findings should be interpreted within the context of certain methodological limitations. Primarily, our evidence for PANoptosis relies largely on protein expression and colocalization analyses. Although these morphological data demonstrate a strong spatial association, they do not directly confirm the physical assembly of a functional PANoptosome complex in vivo [40]. Furthermore, the absence of specific functional assays limits our ability to definitively distinguish among concurrent programmed cell death pathways, including canonical apoptosis, pyroptosis, and necroptosis. As a result, parsing out their exact individual contributions to neuronal loss remains challenging. This reliance on observational data also applies to our investigation of upstream regulators. While our expression profiles phenomenologically implicate ZBP1 in the neuroprotective effects, the current study lacks targeted genetic manipulations, such as ZBP1 knockdown or overexpression, and specific pharmacological inhibitors. Consequently, the necessity and sufficiency of the ZBP1/PANoptosis axis in mediating the effects of AS-IV have yet to be definitively established. To address these gaps, future studies employing rigorous functional validations, targeted genetic interventions, and biochemical structural analyses are needed to unequivocally confirm in vivo PANoptosome formation and its direct mechanistic role in AD.

In summary, with the global prevalence of AD projected to reach 150 million by 2050, discovering novel pathogenic frameworks and effective pharmacological interventions remains an urgent priority [41]. Our study addresses this critical need by bridging the emerging concept of PANoptosis with AD pathology, demonstrating that AS-IV effectively attenuates PANoptosis-associated features in both in vivo and in vitro models. These findings not only offer valuable insights into the integrated cell death networks driving AD progression but also highlight the therapeutic potential of targeting these networks. By broadly modulating concurrent PANoptosis-related processes, AS-IV emerges as a promising pharmacological candidate for future AD treatment.

## 4. Materials and Methods

### 4.1. Chemicals and Reagents

We used the following: Astragaloside IV (221215, Chengdu Kecheng Biotechnology Co., Chengdu, China); Beta-amyloid protein (ab120301, Abcam, Cambridge, UK); Nissl stain (C0117, Beyotime, Shanghai, China). Primary antibodies for Western blot included rabbit anti-IL-18 (10663-1-AP), mouse anti-β-Actin (66009-1-lg), mouse anti-GAPDH (60004-1-lg) (all from Proteintech, Wuhan, China); rabbit anti-CASP3 (AF6370), mouse anti-IgG (H + L) (A0216), rabbit anti-IgG (H + L) (A0208), mouse anti-β-Actin (AF5001) (all from Beyotime); rabbit anti-ZBP1 (IPB9880), rabbit anti-GSDMD (IPB10013), rabbit anti-MLKL (IPH2131) (all from Baijia, Nanjing, China). Antibodies for immunofluorescence included rabbit anti-caspase3 (AF6370), rabbit anti-MLKL (AF1039), rabbit anti-NeuN (AF1072) (all from Beyotime); rabbit anti-GSDMD (IPB10013) (Baijia); goat anti-rabbit IgG (H + L) Cross-Adsorbed Secondary Antibody, HRP (SA00001-2), rabbit anti-fluorescent 488 (SA00013-2), rabbit anti-fluorescent 647 (SA00014-9), and mouse anti-fluorescent 488 (SA00013-1) (all from Proteintech). Tissue autofluorescence quencher (G1221-5ML) and 20× Tris-EDTA antigen retrieval solution (G1203-250ML) were from Servicebio, Wuhan, China.

### 4.2. Animals and Drug Administration

**Figure 7:**
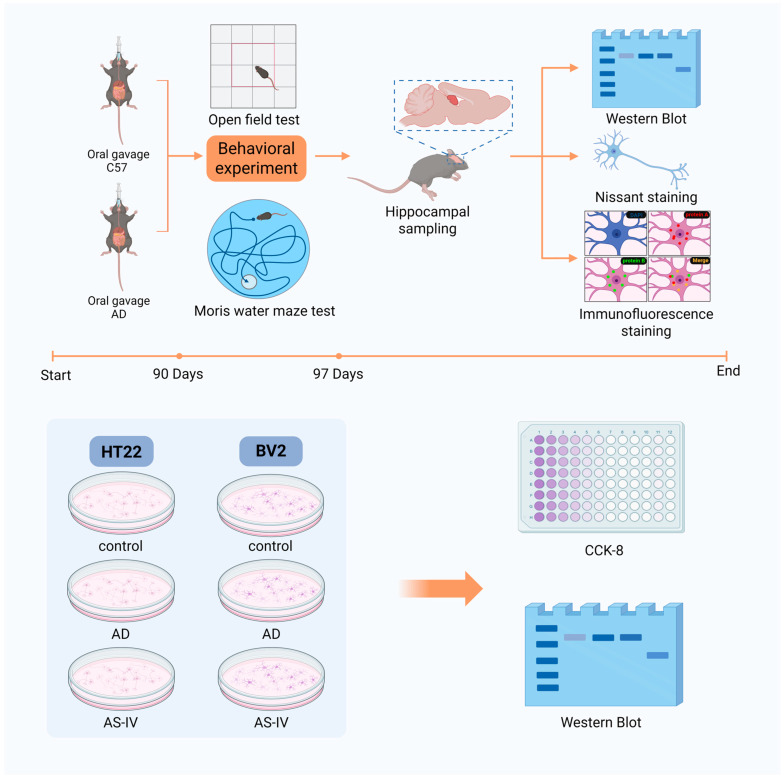
A flowchart of the experimental design. In the in vivo experiments, mice were randomly assigned to three groups (*n* = 10 per group): control, model, and AS-IV treatment groups. The treatment group received Astragaloside IV (AS-IV; 50 mg/kg) by daily oral gavage for 90 consecutive days. Behavioral tests were initiated on the day following the completion of AS-IV administration. After completion of the Morris water maze and open field tests, mice were euthanized, and brain tissues were collected. Nissl staining was performed to evaluate hippocampal neuronal damage. Western blotting was used to quantify the expression of Aβ, IL-18, and PANoptosis-related proteins. Immunofluorescence staining was conducted to assess the co-localization of PANoptosis-associated proteins and their co-localization with neurons. Representative immunofluorescence images show protein A in red, protein B in green, and co-localization in yellow. The timeline illustrates the schedule of the in vivo experiments. For the in vitro studies, the optimal concentration of AS-IV was determined using a CCK-8 assay. BV2 and HT22 cells were divided into control, Aβ, and Aβ + AS-IV groups, treated accordingly, and analyzed by Western blot. Created in BioRender. zhao, Y. (2026) https://BioRender.com/iaqfjos.

All experiments were approved by the Institutional Animal Care and Use Committee of Northeast Forestry University and conducted in accordance with NIH guidelines. 3×Tg-AD mice were purchased from Jackson Laboratory. Animals were housed under specific pathogen-free conditions (temperature: 23 ± 2 °C; humidity: 55 ± 5%; 12 h/12 h light/dark cycle) with sterilized feed, bedding, and water. When all mice reached 8 months of age, experimental intragastric administration was initiated. AS-IV was first dissolved in DMSO and then diluted in saline, and the same concentration of DMSO was included in the vehicle solution administered to the control and AD model groups. Tg mice were assigned to an AD model group or AS-IV treatment groups (12.5, 25, or 50 mg/kg; *n* = 10 per group, except for the 50 mg/kg group, *n* = 20). The AD model group received an equivalent volume of saline (containing the same concentration of DMSO), whereas the AS-IV treatment groups were administered AS-IV once daily by intragastric gavage for 90 consecutive days. Age-matched C57BL/6 mice (Liaoning Changsheng Biotechnology Co., Ltd., Benxi, China) served as controls (*n* = 10) and received an equal volume of vehicle solution. After completion of the intragastric administration period, mice immediately underwent behavioral assessments, including the Morris water maze and open field tests, conducted in a blinded manner, followed by sacrifice for tissue collection (Figure 7). Brain tissues were fixed, embedded in paraffin, and sectioned coronally. The analyzed sections were obtained from the bilateral hippocampal region (Bregma −1.70 mm to −2.80 mm). Nissl staining was performed on 7 μm-thick sections, while immunofluorescence staining was conducted on 5 μm-thick sections. In addition, hippocampal tissues were dissected, homogenized, and subjected to Western blot analysis.

### 4.3. Morris Water Maze Test

The Morris water maze (MWM) is a widely used behavioral test for assessing spatial learning and memory in mice. A circular pool (120 cm in diameter, 50 cm deep) was used, with water temperature maintained at 26 ± 1 °C. The pool was divided into four quadrants of equal area, with distinct visual cues affixed to the wall of each quadrant to facilitate spatial learning and memory. A hidden platform was placed at the center of one quadrant and submerged approximately 1 cm below the water surface. The protocol consisted of a training phase followed by a probe test. During the five-day training phase, mice underwent four trials per day, with the starting position pseudo-randomly assigned to each of the four quadrants and a 5 min inter-trial interval. For each trial, mice were placed into the pool facing the wall at one of four randomized start locations and given 60 s to locate the hidden platform. Mice that failed to find the platform within the allotted time were gently guided to it and allowed to remain there for 10 s. On the day following the completion of the training phase, a probe trial was conducted by removing the platform. Mice were released facing the pool wall from the quadrant opposite to the former platform location and allowed to swim freely for 60 s. Behavioral data were recorded and analyzed using the ANY-maze tracking system. Primary outcome measures included the time spent in the target quadrant (previous platform location) and the number of crossings over the platform’s former position. Total swimming distance and mean swimming speed were also quantified to control for potential motor function impairments.

### 4.4. Open Field Test

Anxiety-like behavior was assessed using the open field test. Prior to testing, mice were placed in an empty cage and allowed to acclimate to the dimly lit experimental environment for 1 h. Each mouse was then gently placed in the corner of a clean, odor-free open field arena (50 × 50 × 50 cm^3^) and allowed to explore freely for 5 min, during which behavior was video-recorded. After each trial, the arena was thoroughly cleaned with 75% ethanol to remove urine and feces and eliminate residual odors before testing the next mouse. The session was recorded and tracked using ANY-MAZE (version 7.4.0.225) software to analyze the total distance traveled, movement speed, and time spent in the central versus peripheral zones. The primary outcomes were the time spent in the central zone and the number of entries into the center.

### 4.5. Cell Culture

BV2 and HT22 cell lines were purchased from the American Type Culture Collection (ATCC, Beijing, China) and cultured in Dulbecco’s Modified Eagle Medium (DMEM) supplemented with 10% fetal bovine serum (FBS) at 37 °C in a 5% CO_2_ atmosphere. Cells were maintained adherently and passaged at 80–90% confluence. For experiments, cells were seeded into 96-well and six-well plates and used at 70–80% confluence.

### 4.6. CCK-8 Assay

Cell viability was assessed using the Cell Counting Kit-8 (CCK-8) assay to determine the cytotoxic effects of AS-IV. Cells were seeded into 96-well plates, and PBS was added to the peripheral wells to minimize medium evaporation. After incubation at 37 °C in a humidified atmosphere containing 5% CO_2_ for 6 h to allow for cell attachment, AS-IV was serially diluted two-fold in culture medium, added to the cells, and incubated for an additional 12 h. Subsequently, 10 µL of CCK-8 reagent was added to each well and incubated at 37 °C for 1 h. Absorbance was measured at 450 nm using a microplate reader (version 2.3).

### 4.7. Cell Treatment

Three experimental groups were established, a control group, an Aβ treatment group, and an Aβ + AS-IV combined treatment group, with repeated samples for each group. The cells were seeded in six-well plates and cultured to 70% confluence. BV2 and HT22 cells were treated with 1 µM and 2 µM AS-IV for 12 h, respectively. The preparation method for Aβ_1–42_ oligomers is as follows: Aβ_1–42_ powder was resuspended in 2 mM sodium hydroxide solution to a concentration of 1 mg/mL. The solution was thoroughly mixed by gently tapping the bottle wall to ensure complete dissolution of the Aβ_1–42_ powder. It was incubated at room temperature for 60 min under closed conditions. The solution was then placed on ice for 5–10 min, transferred, and aliquoted into centrifuge tubes. The caps were opened to allow for complete evaporation of sodium hydroxide, leaving only a clear, transparent peptide film at the bottom of the centrifuge tube. The peptide film was dissolved in DMSO, and sonicated for 30 s to 1 min, gently vortexed to achieve a final concentration of 5 mmol/L. The solution was then diluted with DMEM to 100 μmol/L. After incubation at 4 °C for 24 h, the solution was centrifuged at 14,000× *g* for 10 min in a cold high-speed centrifuge, and the supernatant was collected as the oligomer solution. The oligomer solution was further diluted to the working concentration (10 µM) with medium and applied to the cells for 6 h. After treatment, the cells were collected for Western blot analysis of PANoptosis-related proteins.

### 4.8. Western Blotting

Proteins were extracted from hippocampal tissues and cultured cells using a strong lysis buffer supplemented with protease and phosphatase inhibitors. The tissue samples were homogenized, and the cell lysates were incubated on ice for 30 min with vortexing every 10 min to ensure complete lysis, followed by centrifugation at 4 °C for 15 min. The supernatants were collected, and protein concentrations were determined using a bicinchoninic acid (BCA) assay. After quantification, the protein samples were mixed with 5× loading buffer at a ratio of 4:1 and boiled for 10 min. Then, the protein samples were separated by electrophoresis on 10% SDS–polyacrylamide gels and transferred onto polyvinylidene fluoride (PVDF) membranes. The membranes were blocked with 5% non-fat milk at 37 °C for 90 min and subsequently incubated overnight at 4 °C with primary antibodies against PANoptosis-related proteins (ZBP1, GSDMD, p-MLKL, CASP3, and IL-18) and internal control proteins (β-actin and GAPDH). After washing, the membranes were incubated with species-appropriate horseradish peroxidase (HRP)-conjugated secondary antibodies. Protein bands were visualized using enhanced chemiluminescence and quantified densitometrically using ImageJ software 1.4.3.67. Notably, to address the presence of target proteins with similar molecular weights, a parallel dual-gel electrophoresis strategy was employed. For data processing, the densitometric value of each target protein was strictly normalized to its corresponding internal control (β-actin or GAPDH) derived from the exact same gel prior to any comparative statistical analysis.

### 4.9. Nissl Staining

Paraffin-embedded brain sections were deparaffinized in xylene (2 × 10 min) and rehydrated through a graded ethanol series (100%, 95%, 90%, 80%, 70%, 50%, 30%; 4 min each) to distilled water. Sections were then stained in 0.1% cresyl violet solution at 40 °C for 8–15 min, rinsed gently in distilled water, and differentiated in 95% ethanol. Dehydration was performed through a graded ethanol series (95%, 100%; 4 min each), followed by clearing in xylene (2 × 4 min). Sections were mounted with neutral resin and imaged under a light microscope.

### 4.10. Immunofluorescence

Tissue sections were deparaffinized in xylene (2 × 30 min) and rehydrated through a graded ethanol series (100%, 95%, 90%, 80%, 70%, 50%, and 30%; 4 min each). Antigen retrieval was performed by heating sections in 10 mM sodium citrate buffer (pH 6.0) using a microwave oven (high power for 4 min, then medium–low power for 10 min with 5 min intervals, total 30 min). Endogenous peroxidase activity was quenched with 3% H_2_O_2_ for 25 min at room temperature. Autofluorescence was reduced by incubation with autofluorescence quenching reagent for 30 min. Non-specific binding was blocked with 5% bovine serum albumin (BSA) at room temperature for 1 h. Sections were incubated with primary antibodies (diluted 1:500 in 3% BSA) overnight at 4 °C, followed by incubation with fluorophore-conjugated secondary antibodies (1:200 in 1% BSA) for 1 h at 37 °C in the dark. Nuclei were counterstained with Hoechst 33342 (1:1000 in PBS) for 10 min. Sections were mounted with anti-fade mounting medium and imaged using a fluorescence microscope.

To assess the cells labeled with pan-apoptotic executioner protein antibodies throughout the hippocampus, we collected a large dataset. Images were captured using a Leica Thunder microscope at 20× magnification (Wetzlar, Germany), and the corresponding regions of interest (ROIs) were manually outlined using the polygon selection tool on the ImageJ 1.46r platform for further analysis. To observe the colocalization of pan-apoptotic executioner proteins, fluorescence images were captured at 100× magnification. To indirectly verify the presence of colocalization among the three proteins, we first analyzed the percentage of pairwise colocalization and then inferred the colocalization of the three proteins based on this, further confirming their co-localization within the same cells.

We calculated the ratio of the fluorescence intensity of colocalized signals to the total fluorescence intensity in different regions of the hippocampus. Additionally, the colocalized fluorescence signals were split into independent channels to generate single-channel grayscale images. Background noise was removed using the ROI selection tool. The signals were then normalized based on sample characteristics (such as cell number and total protein content) and noise was removed using Gaussian filtering, median filtering, or wavelet transforms.

### 4.11. Statistical Analysis

Behavioral data were analyzed using ANY-MAZE software. All statistical analyses were performed using GraphPad Prism (version 9.0.0). Data are presented as the mean ± standard error of the mean (SEM). Prior to the analysis, the homogeneity of variances was confirmed using the Brown–Forsythe test and Bartlett’s test. Multiple group comparisons were analyzed by one-way analysis of variance (ANOVA) followed by Dunnett’s post hoc test to determine specific group differences. Escape latency data from the MWM test were analyzed by two-way repeated measures ANOVA, followed by Tukey’s post hoc test for multiple comparisons.

## 5. Conclusions

This study demonstrates that AS-IV treatment improves cognitive performance, preserves neuronal morphology, and simultaneously reduces the expression of multiple PANoptosis-associated proteins in the hippocampus. These observations highlight a close association between PANoptosis-related molecular signatures and AD pathology. Furthermore, they suggest that the neuroprotective benefits of AS-IV are accompanied by the attenuation of these concurrent cell death-related processes, positioning AS-IV as a promising candidate for future therapeutic exploration.

## Data Availability

The original contributions presented in this study are included in the article/Supplementary Materials. Further inquiries can be directed to the corresponding authors.

## Supplementary Materials

The following supporting information can be downloaded at: https://www.mdpi.com/article/10.3390/ijms27083508/s1.

## Abbreviations

AD	Alzheimer’s disease
AS-IV	Astragaloside IV
Aβ	amyloid-β
APP	amyloid precursor protein
AIM2	absent in melanoma 2
BSA	Bovine Serum Albumin
BDNF	brain-derived neurotrophic factor
BV2	BV-2 microglial cell line
CCK-8	Cell Counting Kit-8
CDK9	cyclin dependent kinase 9
CASP3	Cysteine-dependent aspartate-specific protease-3
DMEM	Dulbecco’s Modified Eagle Medium
FBS	fetal bovine serum
GSDMD	Gasdermin D
GSDMD-N	Gasdermin D N-terminal domain
GAPDH	glyceraldehyde-3-phosphate dehydrogenase
HRP	horseradish peroxidase
HT22	Mouse Hippocampal Neuronal Cells
IL-18	Interleukin-18
IκB	inhibitor of NF-κB
MWM	Morris water maze
MLKL	mixed lineage kinase domain-like
Nrf2	Nuclear factor erythroid 2-related factor 2
NFTs	neurofibrillary tangles
NLRP3	nucleotide-binding oligomerization domain-like receptor protein 3
NF-κB	nuclear factor-kappa B
PBS	Phosphate-Buffered Saline
PVDF	polyvinylidene fluoride
p-MLKL	Phospho-mixed lineage kinase domain-like
PPARγ	peroxisome proliferator-activated receptor γ
RIPK1	receptor interacting serine/threonine kinase 1
SEM	standard error of the mean
SOD1	Superoxide Dismutase 1
TrkB	tropomyosin-related kinase B
WB	Western blot
ZBP1	Z-DNA binding protein 1
