# Supplementary material for: Astragaloside IV Improves Cognitive Impairment in Alzheimer’s Mice by Alleviating Neuron PANoptosis
Source: Int J Mol Sci. 2026 Apr 14;27(8):3508. doi: 10.3390/ijms27083508 (PMC13116472; doi:10.3390/ijms27083508)
Supplement: Supplementary file 1 [file ijms-27-03508-s001.zip › ijms-4214884-supplementary.pdf]

This document contains the additional, detailed experimental data that supports the findings reported in the main article. Including this information separately allows the main text to remain concise and focused on the central narrative while making the full dataset available for peer review and for interested readers who want to delve deeper.

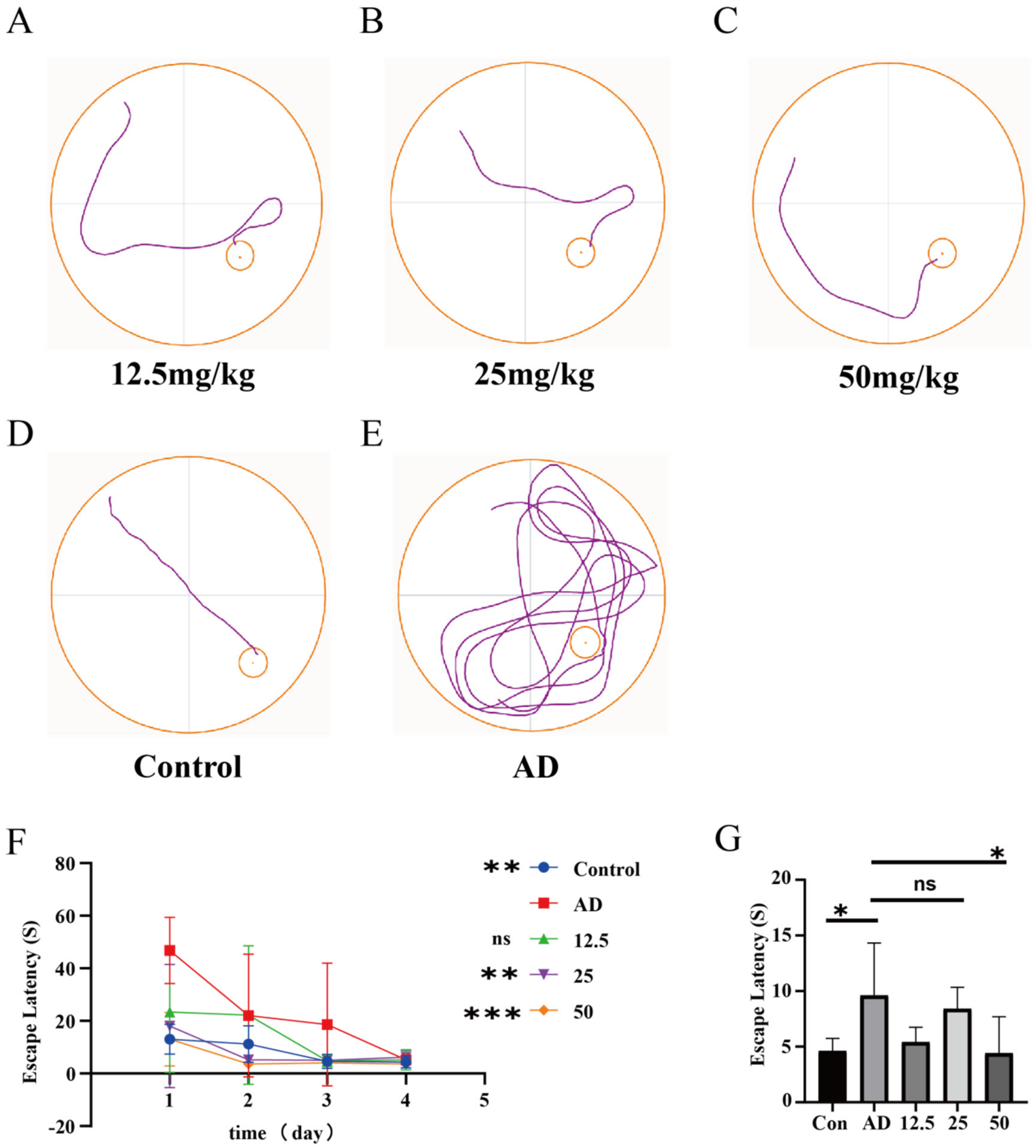

Figure. S1 Water maze experiment(platform visible). (A–E) Movement trajectories of mice administered AS-IV (at doses of 12.5 mg/kg, 25 mg/kg, and 50 mg/kg, as well as those of control and AD model mice. (F) Escape latency across training days. (G) Statistical analysis of escape latency over the training period. n=5. Data are presented as mean ± SEM; *$P < 0.05$, **$P < 0.01$, ***$P < 0.001$.

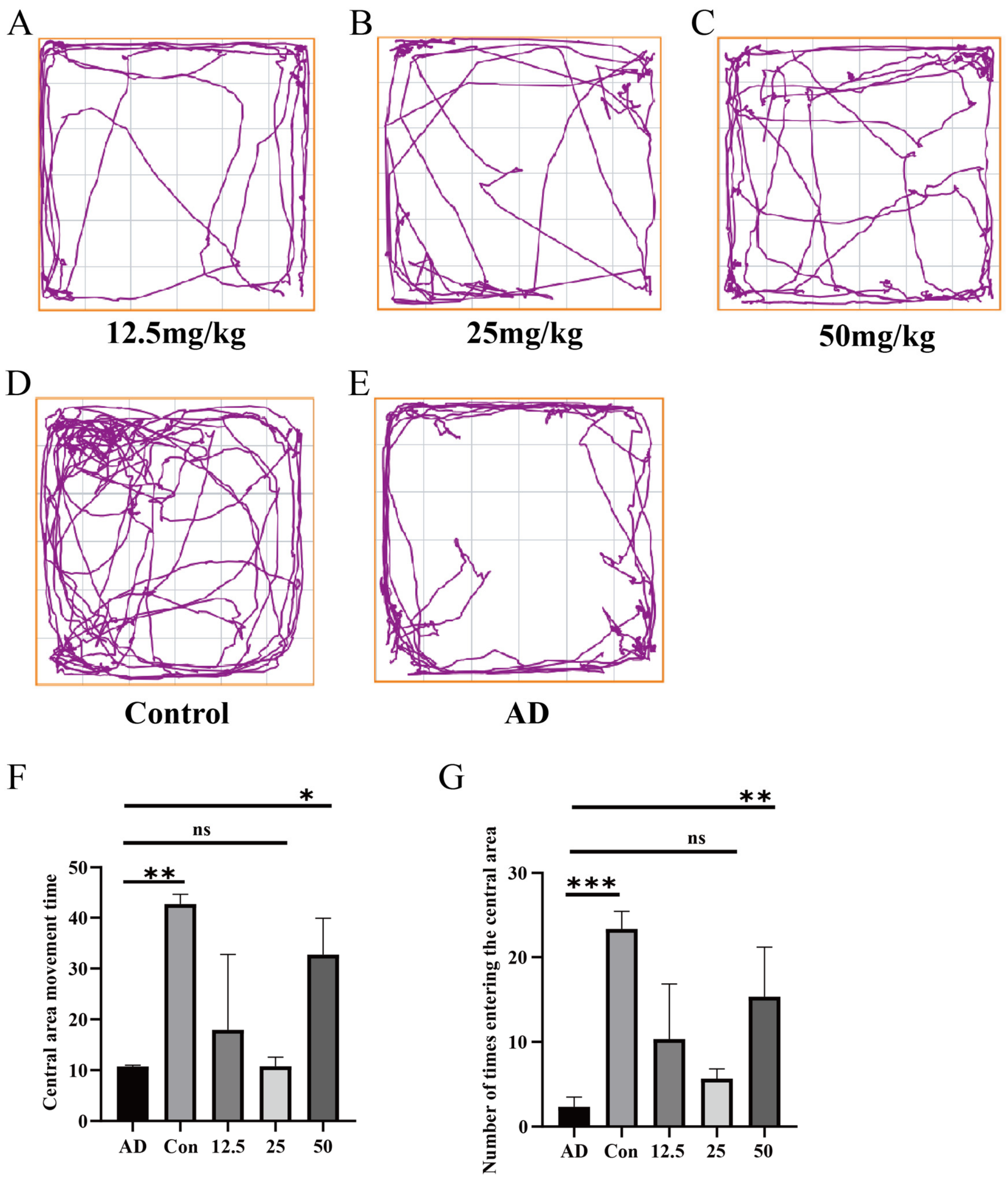

Figure. S2 Open field experiment. (A–E) Movement trajectories of mice administered AS-IV (at doses of 12.5 mg/kg, 25 mg/kg, and 50 mg/kg, as well as those of control and AD model mice. (F) Number of entries into the center zone. (G) Duration spent in the center zone. n=5. Data are presented as mean ± SEM; *P < 0.05, **P < 0.01, ***P < 0.001.

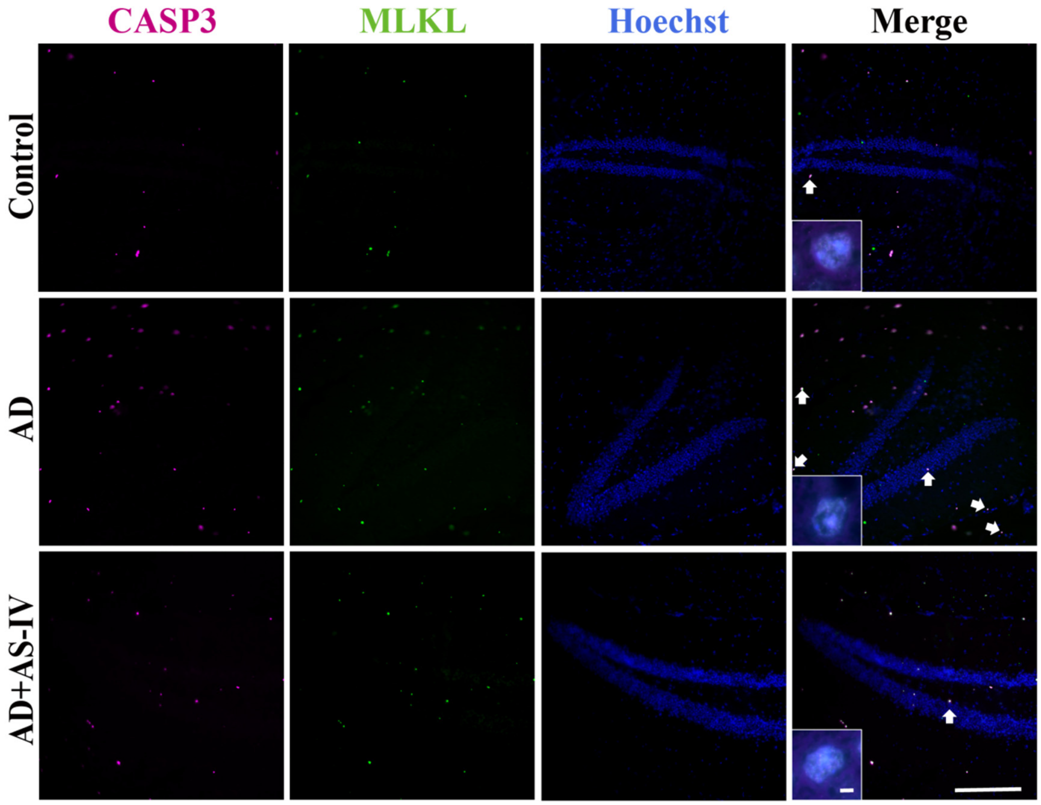

Figure. S3 Immunofluorescence co-localization of CASP3 and MLKL. Scale bar = 100 μm.

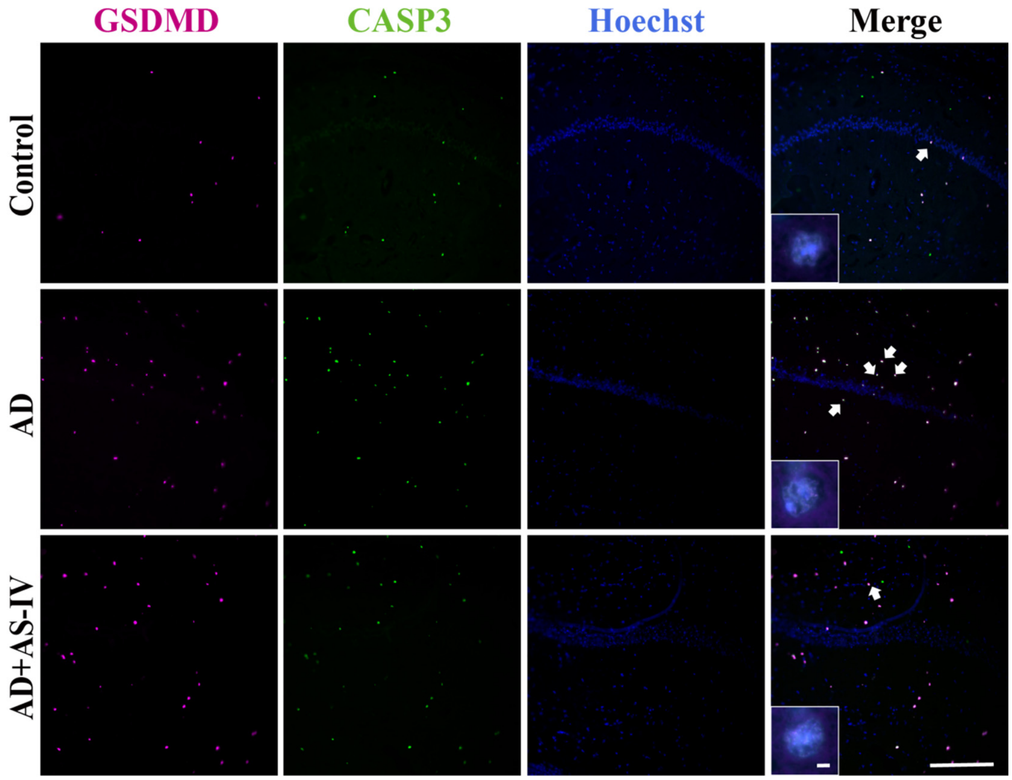

Figure. S4 Immunofluorescence co-localization of GSDMD and CASP3. Scale bar

= 100 μm.

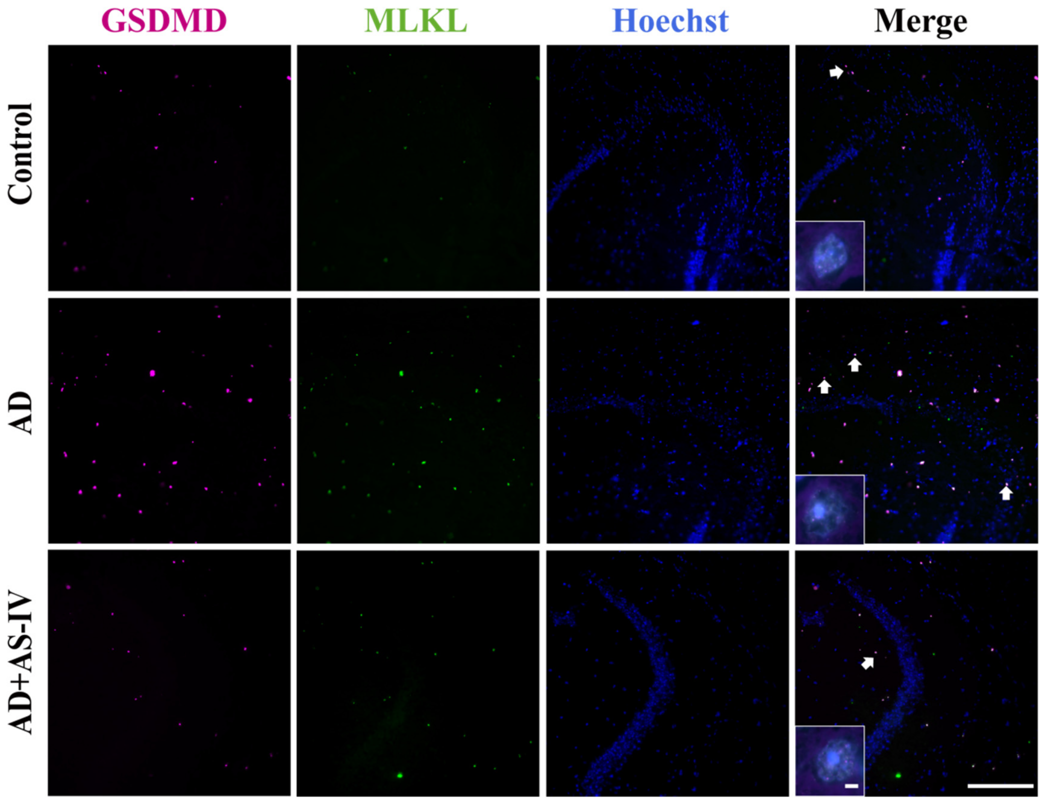

Figure. S5 Immunofluorescence co-localization of GSDMD and MLKL. Scale bar = 100 μm.

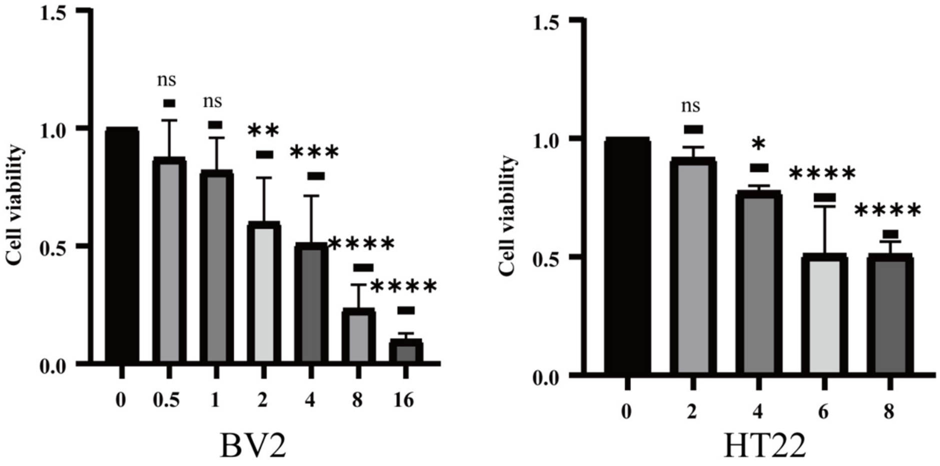

Figure. S6 CCK-8 assay assessing the effect of AS-IV on cell proliferation. (A) BV2 microglial cells. (B) HT22 neuronal cells. n=4. Data are presented as mean ± SEM; *P < 0.05, **P < 0.01, ***P < 0.001, ****P < 0.0001.
